# Fertility Treatments and the Risk of Kawasaki Disease Among Offspring

**DOI:** 10.1001/jamanetworkopen.2026.28586

**Published:** 2026-08-12

**Authors:** Sachiko Baba, Kanami Tanigawa, Junji Miyazaki, Kazuko Wada, Michiko Kodama, Ryo Kawasaki, Hiroyasu Iso

**Affiliations:** 1 Osaka Maternal and Child Information Center, Osaka Women’s and Children’s Hospital, Izumi, Japan; 2 Center for International Relations, Graduate School of Medicine, the University of Osaka, Suita, Japan; 3 Department of Social Medicine, Graduate School of Medicine, the University of Osaka, Suita, Japan; 4 Department of Neonatal Medicine, Osaka Women’s and Children’s Hospital, Izumi, Japan; 5 Department of Obstetrics and Gynecology, Graduate School of Medicine, the University of Osaka, Suita, Japan; 6 Institute for Global Health Policy Research, Bureau of Global Health Cooperation, Japan Institute for Health Security, Tokyo, Japan

## Abstract

**Importance:**

Kawasaki disease is a leading cause of acquired heart disease among children in high-income countries, but potential associations between fertility treatments and Kawasaki disease remain unknown, despite both becoming increasingly prevalent worldwide.

**Objective:**

To examine whether fertility treatments are associated with the risk of Kawasaki disease among offspring before 5 years of age in the general population.

**Design, Setting, and Participants:**

This cohort study used data from the Japan Environmental and Children’s Study, an ongoing nationwide multicenter prospective birth cohort. Pregnant women were recruited between January 2011 and March 2014, and data were analyzed between April and November 2025.

**Exposure:**

Fertility treatments were categorized as spontaneous conception or assisted conception, with the latter further classified into ovulation induction, intrauterine insemination, in vitro fertilization, and intracytoplasmic sperm injection.

**Main Outcomes and Measures:**

The main outcome was Kawasaki disease at up to 59 months of age, reported through parental questionnaires and confirmed by the medical institutions. Hazard ratios (HRs) and 95% CIs were estimated using Cox proportional hazards regression models adjusted for birth year, child’s sex, gestational length at birth, multiple pregnancies, maternal age, older sibling, household income, and area, with additional adjustment for maternal endometriosis and polycystic ovary syndrome, and maternal allergic conditions and autoimmune disease.

**Results:**

During 431 558 person-years of follow-up, 1226 cases of Kawasaki disease were identified among 101 864 children (mean [SD] gestational age, 38.8 [1.7] weeks; 51 402 boys [50.5%]). The incidence rates per 100 000 person-years were 284 overall: 274 for spontaneous conception and 391 for assisted conception. Compared with spontaneous conception, assisted conception was associated with an increased risk of Kawasaki disease (adjusted HR [AHR], 1.42 [95% CI, 1.18-1.70]), as was ovulation induction (AHR, 1.57 [95% CI, 1.22-2.03]), intrauterine insemination (AHR, 1.48 [95% CI, 1.01-2.16]), and in vitro fertilization (AHR, 1.53 [95% CI, 1.07-2.20]), whereas intracytoplasmic sperm injection was not (AHR, 0.91 [95% CI, 0.58-1.43]). These associations were also found after further adjustment for maternal comorbidities.

**Conclusions and Relevance:**

In this nationwide birth cohort study, all fertility treatments except intracytoplasmic sperm injection were associated with an increased risk of Kawasaki disease among offspring. These findings warrant further studies to clarify the underlying mechanisms.

## Key Points

**Question:**

Are fertility treatments associated with an increased risk of Kawasaki disease among offspring?

**Findings:**

In this cohort study of 101 864 Japanese children, ovulation induction, intrauterine insemination, and in vitro fertilization were associated with an increased risk of Kawasaki disease among children at up to 59 months of age compared with spontaneous conception, whereas intracytoplasmic sperm injection was not.

**Meaning:**

Results of this study suggest that most fertility treatments can increase the risk of Kawasaki disease among offspring and that further research is warranted.

## Teaser

This nationwide birth cohort study uses data from the Japan Environmental and Children’s Study to examine the association between fertility treatments and the risk of Kawasaki disease among offspring before 5 years of age in the general population.

## Introduction

Infertility affects approximately 17.5% of couples of reproductive age worldwide.^1^ Various fertility treatments have been developed, ranging from ovulation induction with medications to more complex laboratory-based procedures such as in vitro fertilization and intracytoplasmic sperm injection.^2,3,4^ The use of fertility treatments worldwide has increased markedly over the past several decades.^5,6^ In 2023, approximately 11.7% of all live births in Japan resulted from assisted reproductive technology (ART),^4,7^ which is substantially higher than the rates in Europe (7.9%) and the US (5.1%).^8^ Given this widespread and increasing use of ART, there is also growing interest in understanding the long-term health outcomes of children conceived by these technologies. Previous reports suggest that ART procedures may induce epigenetic modification, including alterations in DNA methylation patterns,^9^ which has been observed in Kawasaki disease (KD).^39^ According to a large birth cohort study in Norway, ART–conceived offspring showed DNA methylation in 176 genes, including genes related to growth, neurodevelopment, and other health outcomes.^10^

KD is a type of systemic vasculitis that primarily affects young children. It is characterized by a severe inflammatory response^11,12^ and was first reported in 1967 by Kawasaki.^13^ Although the exact cause remains unclear, infectious agents are thought to play a role in triggering KD,^12,14^ which remains a leading cause of acquired heart disease in children. The annual incidence rate of KD per 100 000 children aged 59 months or younger in Japan has increased markedly, from 100 in 2001 to 371 in 2019.^15,16^ Approximately 20% of patients not properly treated develop coronary artery aneurysms after KD, leading to lifelong cardiac sequelae.^14,15,17^ Known risk factors for the onset of KD include male sex, age younger than 12 months, presence of siblings, preterm birth, higher socioeconomic status factors, mother’s immune disorders, genetic susceptibility, and certain environmental exposures such as ambient ozone levels and extreme heat.^14,18,19,20,21,22,23^

Despite extensive research on ART and the health of offspring, to our knowledge, no studies have examined whether fertility treatment is associated with KD, both of which are considered to involve immune-related mechanisms and have shown increasing trends.^1,4,14,22^ Therefore, we conducted a nationwide prospective birth cohort study to examine whether conception through various fertility treatments, including ovulation induction, intrauterine insemination, in vitro fertilization, and intracytoplasmic sperm injection, is associated with an increased risk of developing KD during early childhood. We hypothesized that children born after ART would have an increased risk of developing KD.

## Methods

### Study Population and Participants

The present cohort study was based on data obtained from the Japan Environmental and Children’s Study (JECS), an ongoing nationwide prospective birth cohort study that recruited pregnant women between January 2011 and March 2014. Statistical analyses were performed between April and November 2025. Fifteen Regional Centres that covered northern to southern Japan (Hokkaido, Miyagi, Fukushima, Chiba, Kanagawa, Koshin, Toyama, Aichi, Kyoto, Osaka, Hyogo, Tottori, Kochi, Fukuoka, and South Kyushu and Okinawa) were selected. The JECS protocol and profile have been described elsewhere.^24,25^ This study was conducted according to the guidelines in the Declaration of Helsinki.^26^ The JECS protocol was reviewed and approved by the Ministry of the Environment’s Institutional Review Board on Epidemiological Studies and the ethics committees of all participating institutions. Written informed consent was obtained from all participants. This study followed the Strengthening the Reporting of Observational Studies in Epidemiology (STROBE) reporting guideline.

### Confirmation of Kawasaki Disease

KD was included as one of the prespecified pediatric outcomes in the JECS questionnaires from the outset, with parental self-administered questionnaires conducted every 6 months until 6 years of age; questionnaires at ages 6, 12, 18, 24, 36, 48, and 60 months included items regarding KD history, and a secondary survey with the medical institutions listed in the questionnaire confirmed the diagnosis and acquired more detailed information. The cases confirmed through this secondary survey, and with onset up to 59 months of age were included in the present study. Among 1329 confirmed events of KD onset, we excluded recurrent events (n = 46) and those in which the first onset was out of the defined age range (n = 57), resulting in 1226 cases of first-onset KD.

### Exposure

All conceptions were categorized as either spontaneous conception (SC) or assisted conception (AC), which included 4 types of fertility treatments: ovulation induction, intrauterine insemination, in vitro fertilization, and intracytoplasmic sperm injection. These 4 fertility treatment methods fall under the Medical Subject Headings classification of “Reproductive Techniques, Assisted.” Throughout this manuscript, we use the term *AC* or *fertility treatments* to refer collectively to all 4 methods.

Information on fertility treatments was identified through medical record transcripts and self-administered questionnaires filled out by mothers during pregnancy. The former items were transcribed by physicians, nurses, midwives, hospital staff, or research coordinators involved in the JECS. When discrepancies occurred, more advanced treatments were selected in the following descending order of priority: (1) intracytoplasmic sperm injection, (2) in vitro fertilization, (3) intrauterine insemination, (4) ovulation induction, and (5) SC.^27^ If this information was unavailable in the medical record transcripts, “blastocyst transfer” reported by mothers on the questionnaire was classified as in vitro fertilization.^27^ Thus, while in vitro fertilization and intracytoplasmic sperm injection fell under the narrower definition of ART, ovulation induction and intrauterine insemination were categorized as non-ART, as used by the US Centers for Disease Control and Prevention and the American Society for Reproductive Medicine.^2^

### Covariates

The following variables reported to be associated with fertility treatments or KD in previous studies were included as covariates in the present study^8,18,20,21,22,28,29,30^; birth year (2011, 2012, 2013, or 2014, and missing); child’s sex (male or female, and missing); gestational length at birth (preterm, term, or postterm, and missing); multiple pregnancies (yes or no); maternal age at delivery or birth (<25, 25-29, 30-34, 35-39, or ≥40 years, and missing); maternal history of endometriosis (yes or no), polycystic ovary syndrome (yes or no), allergic rhinitis (yes or no), atopic dermatitis (yes or no), asthma (yes or no), and autoimmune diseases (yes or no); older sibling (yes or no); household income (<¥200 000; ¥200 000-¥399 000; ¥400 000-¥599 000; or ≥¥600 000 [to convert to US dollars, multiply by 0.0062]); and area of residence at registration (15 areas based on Regional Centres). The maternal history of these conditions was derived from a self-administered questionnaire completed at study registration. For autoimmune diseases, given the small number of patients with each individual condition recorded (collagen disease, autoimmune disease, systemic lupus erythematosus, rheumatoid arthritis, and other collagen disease or immune-related disease), a composite variable of *autoimmune disease* representing the presence of any autoimmune disease was created. Household income was derived from a self-administered questionnaire during the second and third trimesters. Other items were transcribed from medical records by physicians, midwives, or nurses, and/or research coordinators.

### Statistical Analysis

Descriptive statistics were presented as counts and proportions for all variables. Person-years were calculated from birth to the first end point: the KD onset event or the last questionnaire response, whichever came first. When there was no questionnaire response regarding the child, we set the follow-up period to 1 month if maternal medical transcription data at 1 month post partum was available, or to 0 months if only live birth confirmation was available without any maternal data.

Incidence rates were calculated by dividing the number of cases of KD by the total person-years of follow-up, expressed per 100 000 person-years. Hazard ratios (HRs) and corresponding 95% CIs were calculated using Cox proportional hazards regression models after adjusting for potential confounding factors. To account for clustering of siblings from the same mother, cluster-robust variance estimation by mother ID was applied to all models. In model 1, we adjusted for birth year, child’s sex, gestational length at birth, multiple pregnancies, maternal age, older sibling, household income, and area. In model 2, we further adjusted for maternal endometriosis and maternal polycystic ovary syndrome, and in model 3 we additionally adjusted for maternal history of allergic rhinitis, atopic dermatitis, asthma, and autoimmune disease. For exposure variables, we calculated HRs for AC using SC as the reference and for fertility treatment (ovulation induction, intrauterine insemination, in vitro fertilization, and intracytoplasmic sperm injection) with SC as the reference.

The assumption in the Cox proportional hazards regression analysis was assessed by visual inspection of log (-log [survival]) plots and by examination of scaled Schoenfeld residuals, and it was not violated. The same analytical methods were applied as in the main analysis. Missing values were included as dummy variables. Tests of statistical significance were based on 2-tailed *P* values or 95% CIs, and statistical significance was set at *P* < .05. SAS software, version 9.4 (SAS Institute Inc) was used for the statistical analyses.

## Results

Among 104 043 pregnancies, there were 102 407 live births. We excluded those with missing information on the conception method (n = 543), leaving a final sample size of 101 864 (eFigure in Supplement 1). Among the 101 864 participants (mean [SD] gestational age, 38.8 [1.7] weeks; 51 402 boys [50.5%] and 48 864 girls [48.0%]), 93 484 (91.8%) were born from SC and 8380 (8.2%) from AC, which were further divided into 3381 cases of ovulation induction (3.3% of all participants), 1536 cases of intrauterine insemination (1.5%), 1619 cases of in vitro fertilization (1.6%), 1798 cases of intracytoplasmic sperm injection (1.8%), and 46 cases of ART with missing details (0.1%) (Table 1). AC (either non-ART or ART) was associated with preterm births, multiple births, and older mothers. Those who underwent non-ART, particularly ovulation induction, were more likely to have polycystic ovary syndrome, whereas those who underwent ART (in vitro fertilization and intracytoplasmic sperm injection) were more likely to have endometriosis and higher household income compared with those who underwent SC.

**Table 1.** Descriptive Characteristics of Study Participants

Characteristic	No. (%)		AC, No. (%)				
	Total (N = 101 864)	SC (n = 93 484)	Non-ART (n = 4917)		ART (n = 3463)		
			Ovulation induction (n = 3381)	Intrauterine insemination (n = 1536)	In vitro fertilization (n = 1619)	Intracytoplasmic sperm injection (n = 1798)	Detail missing (n = 46)
Birth year							
2011	9743 (9.6)	9094 (9.7)	299 (8.8)	90 (5.9)	129 (8.0)	128 (7.1)	3 (6.5)
2012	28 276 (27.8)	26 095 (27.9)	880 (26.0)	405 (26.4)	415 (25.6)	468 (26.0)	13 (28.3)
2013	35 615 (35.0)	32 525 (34.8)	1278 (37.8)	568 (37.0)	581 (35.9)	652 (36.3)	11 (23.9)
2014	26 650 (26.2)	24 291 (26.0)	884 (26.1)	452 (29.4)	482 (29.8)	529 (29.4)	12 (26.1)
Missing	1580 (1.6)	1479 (1.6)	40 (1.2)	21 (1.4)	12 (0.7)	21 (1.2)	7 (15.2)
Child’s sex							
Male	51 402 (50.5)	47 175 (50.5)	1709 (50.5)	755 (49.2)	859 (53.1)	883 (49.1)	21 (45.7)
Female	48 864 (48.0)	44 812 (47.9)	1632 (48.3)	760 (49.5)	748 (46.2)	894 (49.7)	18 (39.1)
Missing	1598 (1.6)	1497 (1.6)	40 (1.2)	21 (1.4)	12 (0.7)	21 (1.2)	7 (15.2)
Gestational length							
Preterm	5579 (5.5)	4740 (5.1)	333 (9.8)	127 (8.3)	184 (11.4)	190 (10.6)	5 (10.9)
Term	94 150 (92.4)	86 767 (92.8)	2985 (88.3)	1378 (89.7)	1413 (87.3)	1573 (87.5)	34 (73.9)
Postterm	226 (0.2)	196 (0.2)	7 (0.2)	5 (0.3)	7 (0.4)	11 (0.6)	0
Missing	1909 (1.9)	1781 (1.9)	56 (1.7)	26 (1.7)	15 (0.9)	24 (1.3)	7 (15.2)
Multiple pregnancies	1889 (1.9)	1216 (1.3)	282 (8.3)	113 (7.4)	138 (8.5)	138 (7.7)	2 (4.3)
Mother’s age at birth, y							
<25	10 038 (9.9)	9953 (10.6)	76 (2.2)	5 (0.3)	0	2 (0.1)	2 (4.3)
25-29	27 677 (27.2)	26 478 (28.3)	852 (25.2)	150 (9.8)	85 (5.3)	110 (6.1)	2 (4.3)
30-34	35 457 (34.8)	32 547 (34.8)	1422 (42.1)	546 (35.5)	448 (27.7)	485 (27.0)	9 (19.6)
35-39	22 537 (22.1)	19 424 (20.8)	861 (25.5)	645 (42.0)	775 (47.9)	812 (45.2)	20 (43.5)
≥40	4598 (4.5)	3624 (3.9)	132 (3.9)	169 (11.0)	299 (18.5)	368 (20.5)	6 (13.0)
Missing	1557 (1.5)	1458 (1.6)	38 (1.1)	21 (1.4)	12 (0.7)	21 (1.2)	7 (15.2)
Mother’s endometriosis	3653 (3.6)	2855 (3.1)	204 (6.0)	132 (8.6)	226 (14.0)	232 (12.9)	4 (8.7)
Mother’s polycystic ovary syndrome	2296 (2.3)	1182 (1.3)	636 (18.8)	168 (10.9)	156 (9.6)	153 (8.5)	1 (2.2)
Mother’s allergic rhinitis	35 918 (35.3)	32 812 (35.1)	1233 (36.5)	586 (38.2)	570 (35.2)	707 (39.3)	10 (21.7)
Mother’s atopic dermatitis	15 677 (15.4)	14 392 (15.4)	522 (15.4)	253 (16.5)	256 (15.8)	251 (14.0)	3 (6.5)
Mother’s asthma	10 899 (10.7)	10 103 (10.8)	326 (9.6)	163 (10.6)	155 (9.6)	148 (8.2)	4 (8.7)
Any collagen or autoimmune disease	661 (0.6)	579 (0.6)	27 (0.8)	18 (1.2)	17 (1.1)	20 (1.1)	0
Older sibling	54 784 (53.8)	51 986 (55.6)	1351 (40.0)	422 (27.5)	489 (30.2)	514 (28.6)	22 (47.8)
Household income, ¥^a^							
<200 000	5190 (5.1)	5056 (5.4)	65 (1.9)	24 (1.6)	24 (1.5)	19 (1.1)	2 (4.3)
200 000-399 000	31 641 (31.1)	29 903 (32.0)	850 (25.1)	301 (19.6)	291 (18.0)	289 (16.1)	7 (15.2)
400 000-599 000	30 289 (29.7)	27 558 (29.5)	1135 (33.6)	510 (33.2)	524 (32.4)	554 (30.8)	8 (17.4)
≥600 000	24 528 (24.1)	21 385 (22.9)	1065 (31.5)	582 (37.9)	668 (41.3)	813 (45.2)	15 (32.6)
Missing	10 216 (10.0)	9582 (10.2)	266 (7.9)	119 (7.7)	112 (6.9)	123 (6.8)	14 (30.4)

Abbreviations: AC, assisted conception (fertility treatments); ART, artificial reproductive technique; SC, spontaneous conception.

^a^ To convert to US dollars, multiply by 0.0062.

During the 431 558 person-years of follow-up for the 101 864 participants, there were a total of 1226 cases of KD before 5 years (≤59 months) of age (Table 2). The incidence rates of KD per 100 000 person-years were 284 overall: 274 for SC and 391 for AC. Compared with SC, AC was associated with an increased risk of KD after adjusting for covariates in model 1 (adjusted HR [AHR], 1.42 [95% CI, 1.18-1.70]). When AC was divided into non-ART and ART, the corresponding incidence rates per 100 000 person-years and adjusted HRs compared with SC were 429 (AHR, 1.54 [95% CI, 1.24-1.92]) for non-ART and 340 (AHR, 1.23 [95% CI, 0.92-1.63]) for ART. When further divided, the incidence rates per 100 000 person-years were 437 for ovulation induction, 410 for intrauterine insemination, 438 for in vitro fertilization, and 246 for intracytoplasmic sperm injection. Ovulation induction (AHR, 1.57 [95% CI, 1.22-2.03]), intrauterine insemination (AHR, 1.48 [95% CI, 1.01-2.16]), and in vitro fertilization (AHR, 1.53 [95% CI, 1.07-2.20]) were associated with an increased risk of KD compared with SC, whereas intracytoplasmic sperm injection was not associated with the risk of KD- (AHR, 0.91 [95% CI, 0.58-1.43]). In model 2, which was further adjusted for endometriosis and polycystic ovary syndrome, the increased risks did not change; the AHR for ovulation induction was 1.55 (95% CI, 1.19-2.01), for intrauterine insemination was 1.46 (95% CI, 1.00-2.15), and for in vitro fertilization was 1.52 (95% CI, 1.06-2.19). In model 3, which was further adjusted for maternal allergic conditions and autoimmune disease, similar patterns were observed; the AHR for ovulation induction was 1.56 (95% CI, 1.19-2.03), for intrauterine insemination was 1.41 (95% CI, 0.95-2.09), and for in vitro fertilization was 1.55 (95% CI, 1.08-2.22).

**Table 2.** Incidence Rates and Hazard Ratios of Kawasaki Disease During Ages ≤59 Months, by Method of Conception

Characteristic	No. at risk (n = 101 864)	Person-years (n = 431 558)	Incidence rate per 100 000 person-years (n = 284)	No. of cases (n = 1226)	Adjusted hazard ratio (95%CI)		
					Model 1^a^	Model 2^b^	Model 3^c^
Spontaneous conception	93 484	394 258	274	1080	1 [Reference]	1 [Reference]	1 [Reference]
Assisted conception	8380	37 300	391	146	1.42 (1.18-1.70)	1.40 (1.15-1.69)	1.40 (1.15-1.69)
Non-ART	4917	21 702	429	93	1.54 (1.24-1.92)	1.52 (1.21-1.91)	1.51 (1.20-1.90)
Ovulation induction	3381	14 875	437	65	1.57 (1.22-2.03)	1.55 (1.19-2.01)	1.56 (1.19-2.03)
Intrauterine insemination	1536	6827	410	28	1.48 (1.01-2.16)	1.46 (1.00-2.15)	1.41 (0.95-2.09)
ART	3463	15 598	340	53	1.23 (0.92-1.63)	1.22 (0.91-1.62)	1.23 (0.92-1.64)
In vitro fertilization	1619	7305	438	32	1.53 (1.07-2.20)	1.52 (1.06-2.19)	1.55 (1.08-2.22)
Intracytoplasmic sperm injection	1798	8125	246	20	0.91 (0.58-1.43)	0.91 (0.58-1.42)	0.91 (0.58-1.42)
Missing	46	168	595	1	2.03 (0.28-14.60)	2.03 (0.28-14.61)	2.41 (0.34-17.15)

Abbreviation: ART, artificial reproductive technique.

^a^ Adjusted for birth year, mother’s age, multiple pregnancies, gestational age at birth, child’s sex, older sibling, household income, and area.

^b^ Adjusted further for mother’s endometriosis and polycystic ovary syndrome.

^c^ Adjusted further for mother’s allergic rhinitis, atopic dermatitis, asthma, and autoimmune disease.

## Discussion

In the present nationwide prospective birth cohort study, fertility treatments were associated with an increased risk of KD until 59 months of age after adjusting for potential confounding factors such as birth year, child sex, and perinatal and maternal factors. Increased risks were similarly observed among ovulation induction, intrauterine insemination, and in vitro fertilization, but not intracytoplasmic sperm injection, and did not change materially after additional adjustment for maternal endometriosis and polycystic ovary syndrome.

The observed incidence rate of KD in this study of children born in 2011 to 2014 (284 per 100 000 person-years) was slightly lower than the corresponding incidence reported by a hospital-based study from Japan (1444 pediatric departments in hospitals with ≥100 beds) conducted in 2015 and 2016 (309 per 100 000 population per year),^31^ although the data from the 2 studies are not strictly comparable because of the difference in sampling.

To our knowledge, this is the first study to assess whether fertility treatments are associated with KD among offspring. We found that, with the exception of intracytoplasmic sperm injection, fertility treatments were associated with increased risks of KD among children. The potential biological mechanisms for our findings are as follows. First, the ovarian stimulation used in most fertility treatments creates a supraphysiological hormonal environment during oocyte maturation and early embryonic development.^32,33^ This hormonal environment has been reported to perturb 1-carbon cycle metabolism, leading to homocysteine accumulation in follicular fluid, a potent inhibitor of DNA methyltransferase activity.^34^ Such disruption of methylation during early embryonic development may induce epigenetic predisposition in offspring.

Second, growing evidence suggests that such ART procedures induce epigenetic modifications, including altered methylation patterns at imprinted genes in offspring, which may affect immune system development and its function.^35,36,37^ In KD, hypomethylation due to immune regulatory genes including toll-like receptor family (*TLR1*, *TLR2*, *TLR4*, *TLR6*, *TLR8*, and *TLR9*) and *FCGR2A* was found in the acute phase, with upregulated expression of these genes leading the systemic inflammation.^38,39^ KD is believed to result from an aberrant immune response to infectious triggers in genetically susceptible individuals,^14^ and altered immune programming due to epigenetic modifications induced by ART could increase disease susceptibility.

Increased risks of KD were observed among children conceived through conventional in vitro fertilization, intrauterine insemination, and ovulation induction, but not intracytoplasmic sperm injection. This differential association may be explained by the underlying cause of infertility. Conventional in vitro fertilization, intrauterine insemination, and ovulation induction are more commonly used for female factor infertility, including endometriosis and polycystic ovary syndrome,^40,41^ whereas intracytoplasmic sperm injection is primarily indicated for male factor infertility.^42^ Another consideration is that intracytoplasmic sperm injection involves artificial sperm selection and mechanical puncture of the oocyte membrane, whereas sperm penetrate the oocyte naturally without direct manipulation of the intracellular environment in conventional in vitro fertilization.^42,43^ This procedural difference could theoretically result in different consequences, although the specific mechanisms remain unclear. Our finding that the increased risk of KD was significant even after adjusting for maternal endometriosis and polycystic ovary syndrome suggests that the association is not entirely explained by the maternal conditions that led to infertility, but rather that the fertility treatment procedures themselves or other unmeasured aspects of subfertility may play a role.

### Strengths and Limitations

This study has several strengths, including its nationwide prospective birth cohort design and long follow-up periods. The large sample size provided adequate statistical power to detect small effect sizes and examine different types of fertility treatments separately. Data collection through both medical record transcripts and parental self-reports enhanced the accuracy of our fertility treatment classifications. The use of a comprehensive national database minimized selection bias and ensured generalizability to the broader population. Furthermore, the ability to adjust for important maternal factors, including underlying infertility diagnoses and socioeconomic factors, strengthened the internal validity of our findings.

However, several limitations should be acknowledged. First, despite the large overall sample size, statistical power may have been insufficient to detect associations in smaller subgroups, such as intracytoplasmic sperm injection–conceived children. Second, the possibility of some misclassification of fertility treatment methods cannot be excluded, despite the use of medical record data. In addition, information on specific treatment protocols, including details of ovarian stimulation regimens, culture media compositions, and embryo quality, was not available in our dataset. Third, some cases may have been undetected if they were not reported by the parents in the questionnaires. Fourth, we did not account for unmeasured variables, including genetic factors.

## Conclusions

In this nationwide cohort study, most fertility treatments, with the exception of intracytoplasmic sperm injection, were associated with an increased risk of KD among offspring. Further research is necessary to examine the consistency of our findings and elucidate the underlying mechanisms.

## Notes

Supplement 1. **eFigure.** Flowchart of the Selection Process of Study Participants

Supplement 2. **Nonauthor Collaborators.** Japan Environmental and Children’s Study Group members

Supplement 3.
Data Sharing Statement
