# Supplementary material for: Fertility Treatments and the Risk of Kawasaki Disease Among Offspring
Source: JAMA Netw Open. 2026 Aug 12;9(8):e2628586. doi: 10.1001/jamanetworkopen.2026.28586 (PMC13470285; doi:10.1001/jamanetworkopen.2026.28586)
Supplement: Supplementary file 1 [file jamanetwopen-e2628586-s001.pdf]

Supplementary Online Content

Baba S, Tanigawa K, Miyazaki J, et al. Fertility treatments and the risk of Kawasaki disease among offspring. *JAMA Netw Open.* 2026;9(8):e2628586. doi:10.1001/jamanetworkopen.2026.28586

eFigure. Flowchart of the Selection Process of Study Participants

This supplementary material has been provided by the authors to give readers additional information about their work.

© 2026 Baba S et al. *JAMA Network Open.*

eFigure. Flowchart of the Selection Process of Study Participants

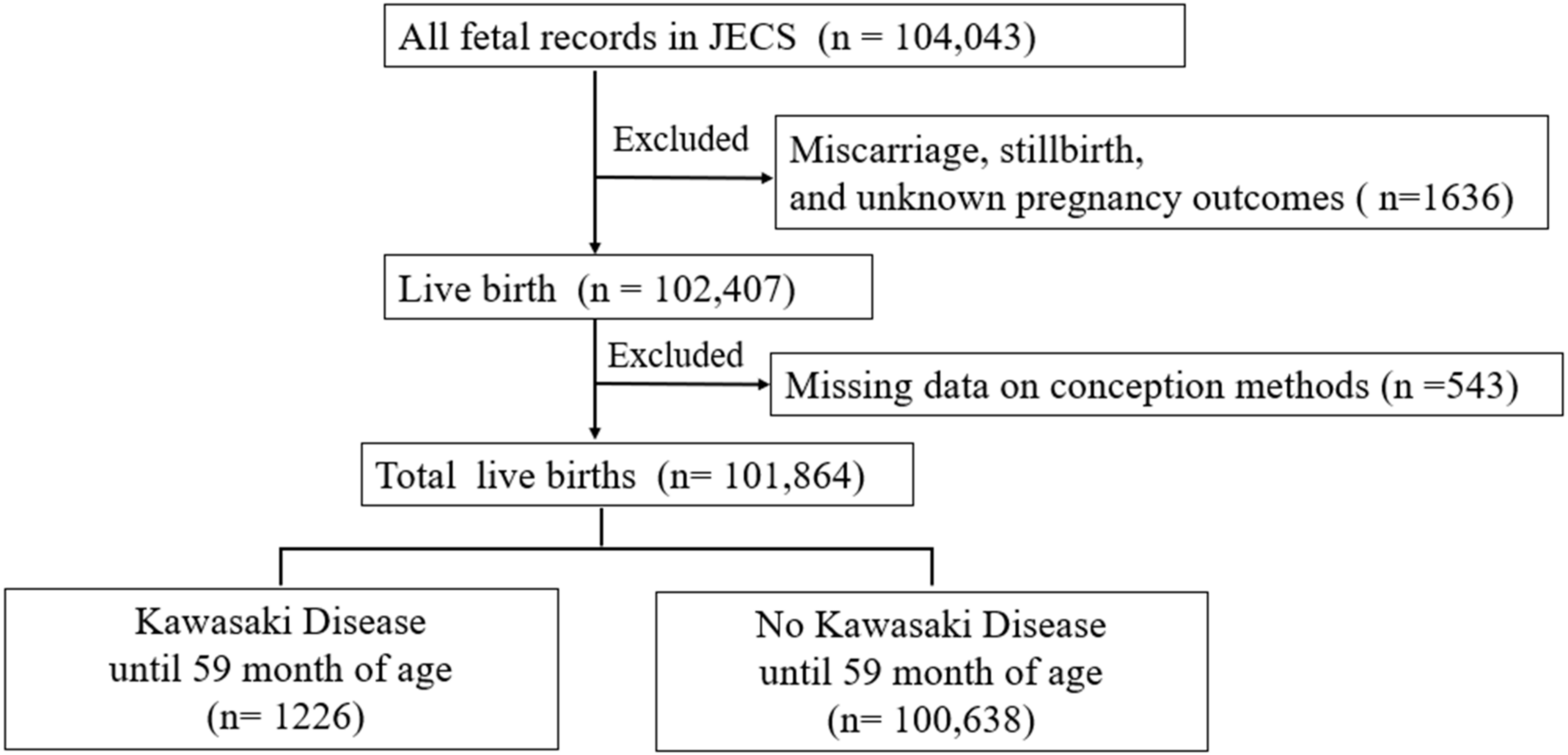

© 2026 Baba S et al. *JAMA Network Open.*
