# Supplementary material for: Fertility Treatments and the Risk of Kawasaki Disease Among Offspring
Source: JAMA Netw Open. 2026 Aug 12;9(8):e2628586. doi: 10.1001/jamanetworkopen.2026.28586 (PMC13470285; doi:10.1001/jamanetworkopen.2026.28586)
Supplement: Supplementary file 2 [file jamanetwopen-e2628586-s002.pdf]

*First name, last name, and suffix (if applicable) are required and will appear in PubMed.

***Group Name(s): The Japan Environment and Children's Study Group**										
***First Name and Middle Initial(s)**	***Last Name**	***Suffix (eg, Jr, III)**	Academic Degrees	Institution	Location (city, state/province, country)	Role or Contribution, eg, chair, principal investigator	Group (if more than 1 Group listed in the byline) and/or Subgroup (eg, Steering Committee)			
Michihiro	Kamijima		MD, PhD	Nagoya City University	Nagoya, Japan	Principal investigator				
Shin	Yamazaki		DrPH	National Institute for Environmental	Tsukuba, Japan	Programme Office	Programme Office Director			
Maki	Fukami		MD, PhD	National Center for Child Health and	Tokyo, Japan	Medical Support Centr	Medical Support Centre Director			
Reiko	Kishi		MD, PhD, MPH	Hokkaido University	Sapporo, Japan	Hokkaido Regional Ce	Regional Centre Director			
Chiharu	Ota		MD, PhD	Tohoku University	Sendai, Japan	Miyagi Regional Cente	Regional Centre Director			
Koichi	Hashimoto		MD, PhD	Fukushima Medical University	Fukushima, Japan	Fukushima Regional C	Regional Centre Director			
Kenichi	Sakurai		MD, PhD	Chiba University	Chiba, Japan	Chiba Regional Centre	Regional Centre Director			
Shuichi	Ito		MD, PhD	Yokohama City University	Yokohama, Japan	Kanagawa Regional C	Regional Centre Director			
Ryoji	Shinohara		MD, PhD	University of Yamanashi	Chuo, Japan	Koshin Regional Centr	Regional Centre Director			
Hidekuni	Inadera		MD, PhD	University of Toyama	Toyama, Japan	Toyama Regional Cent	Regional Centre Director			
Takeo	Nakayama		MD, PhD	Kyoto University	Kyoto, Japan	Kyoto Regional Centre	Regional Centre Director			
Ryo	Kawasaki		MD, PhD, MPH	Osaka University	Suita, Japan	Osaka Regional Centre	Regional Centre Director			
Yasuhiro	Takeshima		MD, PhD	Hyogo Medical University	Nishinomiya, Japan	Hyogo Regional Centr	Regional Centre Director			
Hideki	Nagashima		MD, PhD	Tottori University	Yonago, Japan	Tottori Regional Centr	Regional Centre Director			
Narufumi	Suganuma		MD, PhD	Kochi University	Nankoku, Japan	Kochi Regional Centre	Regional Centre Director			
Mayumi	Tsuji		MD, PhD	University of Occupational and Er	Kitakyushu, Japan	Fukuoka Regional Cen	Regional Centre Director			
Kimitoshi	Nakamura		MD, PhD	Kumamoto University	Kumamoto, Japan	South Kyusyu/Okinaw	Regional Centre Director			
Sayaka	Fukuda		MD, PhD	Yokohama City University	Yokohama, Japan	Kanagawa Regional C	Kawasaki Disease Medical Record Transcription Board			
Tohru	Kobayashi		MD, PhD	Yokohama City University	Yokohama, Japan	Kanagawa Regional C	Kawasaki Disease Medical Record Transcription Board			
