# Supplementary material for: Fertility Treatments and the Risk of Kawasaki Disease Among Offspring
Source: JAMA Netw Open. 2026 Aug 12;9(8):e2628586. doi: 10.1001/jamanetworkopen.2026.28586 (PMC13470285; doi:10.1001/jamanetworkopen.2026.28586)
Supplement: Supplementary file 3 [file jamanetwopen-e2628586-s003.pdf]

Data Sharing Statement

Baba. Fertility Treatments and the Risk of Kawasaki Disease Among Offspring. *JAMA Netw Open*. Published August 12, 2026. doi:10.1001/jamanetworkopen.2026.28586

Data
Data available: No

Additional Information
Explanation for why data not available: Data are unsuitable for public deposition due to ethical restrictions and legal framework of Japan. It is prohibited by the Act on the Protection of Personal Information (Act No. 57 of 30 May 2003, amendment on 9 September 2015) to publicly deposit the data containing personal information. Ethical Guidelines for Medical and Health Research Involving Human Subjects enforced by the Japan Ministry of Education, Culture, Sports, Science and Technology and the Ministry of Health, Labour and Welfare also restricts the open sharing of the epidemiologic data. All inquiries about access to data should be sent to: jecs-en@nies.go.jp. The person responsible for handling enquiries sent to this e-mail address is Dr Shoji F. Nakayama, JECS Programme Office, National Institute for Environmental Studies.
